# Improving Participant Recruitment in Clinical Trials: Comparative Analysis of Innovative Digital Platforms

**DOI:** 10.2196/60504

**Published:** 2024-12-18

**Authors:** Alexia Georgia Bikou, Elena Deligianni, Foteini Dermiki-Gkana, Nikolaos Liappas, José Gabriel Teriús-Padrón, Maria Eugenia Beltrán Jaunsarás, Maria Fernanda Cabrera-Umpiérrez, Christos Kontogiorgis

**Affiliations:** 1 Department of Medicine Democritus University of Thrace Alexandroupolis Greece; 2 Life Supporting Technologies (LifeSTech) Superior Technical School of Telecommunication Engineers Universidad Politécnica de Madrid (UPM) Madrid Spain

**Keywords:** clinical research, e-recruitment, patient matching, clinical trials, digital platforms, enrollment, electronic consent

## Abstract

**Background:**

Pharmaceutical product development relies on thorough and costly clinical trials. Participant recruitment and monitoring can be challenging. The incorporation of cutting-edge technologies such as blockchain and artificial intelligence has revolutionized clinical research (particularly in the recruitment stage), enhanced secure data storage and analysis, and facilitated participant monitoring while protecting their personal information.

**Objective:**

This study aims to investigate the use of novel digital platforms and their features, such as e-recruitment, e-consent, and matching, aiming to optimize and expedite clinical research.

**Methods:**

A review with a systematic approach was conducted encompassing literature from January 2000 to October 2024. The MEDLINE, ScienceDirect, Scopus, and Google Scholar databases were examined thoroughly using a customized search string. Inclusion criteria focused on digital platforms involving clinical trial recruitment phases that were in English and had international presence, scientific validation, regulatory approval, and no geographic limitations. Literature reviews and unvalidated digital platforms were excluded. The selected studies underwent meticulous screening by the research team, ensuring a thorough analysis of novel digital platforms and their use and features for clinical trials.

**Results:**

A total of 24 digital platforms were identified that supported clinical trial recruitment phases. In general, most of them (n=22, 80%) are headquartered and operating in the United States, providing a range of functionalities including electronic consent (n=14, 60% of the platforms), participant matching, and monitoring of patients’ health status. These supplementary features enhance the overall effectiveness of the platforms in facilitating the recruitment process for clinical trials. The analysis and digital platform findings refer to a specific time frame when the investigation took place, and a notable surge was observed in the adoption of these novel digital tools, particularly following the COVID-19 outbreak.

**Conclusions:**

This study underscores the vital role of the identified digital platforms in clinical trials, aiding in recruitment, enhancing patient engagement, accelerating procedures, and personalizing vital sign monitoring. Despite their impact, challenges in accessibility, compatibility, and transparency require careful consideration. Addressing these challenges is crucial for optimizing digital tool integration into clinical research, allowing researchers to harness the benefits while managing the associated risks effectively.

## Introduction

### Background

Developing a new pharmaceutical product or revising and indicating potential new uses for existing products is a meticulous, time-consuming, and costly process [1]. Clinical trials ultimately represent a key tool in clinical research for advancing medical scientific knowledge and patient care [2]. Recruiting the appropriate target and number of participants remains one of the significant challenges in clinical trials [3]. The recruitment stage is a critical factor in ensuring the success and quality of a clinical trial [4,5]. Recent statistics now indicate that approximately 80% of clinical trials face delays due to patient recruitment issues [6]. Many of these delays last between 1 and 6 months, with some trials facing even longer setbacks. In addition, a significant number of research sites enroll very few or no patients, worsening the delays [6]. The success of clinical trials heavily depends on the prompt identification and recruitment of individuals meeting the study’s inclusion criteria [7]. Over the years, a face-to-face approach has been used in various stages of clinical trial research, including recruitment, consent for enrollment, retention, delivery of interventions, and data collection [8,9]. However, it has been widely observed that a significant number of individuals and patients interested in participating in a clinical trial are not adequately informed about how to identify and approach clinical trials that align with their needs and interests [10]. In addition, numerous factors such as the number of patients to be screened, the participating sites, clinical institutions’ infrastructures, and data protection laws may significantly affect recruitment [11]. The lack of accessibility to funds and the high costs associated with conducting clinical trials are also notable barriers to the recruitment stage and the overall trial processes [12]. Despite the performance of clinical trials worldwide, the time-consuming manual process of selecting the participants and matching them to the right study remains a major challenge in the trial recruitment stage [13]. Given the increasing number of accessible clinical trials and the complexity of their designs, the aforementioned processes demand in-depth understanding of patient characteristics and eligibility requirements based on the inclusion criteria [14].

In recent years, the integration of cutting-edge technologies into clinical trials has significantly influenced the successful and expedited conduct of various clinical trial phases, especially the recruitment process [15,16]. The expansion of web access; computational tools; and portable devices such as smartphones, tablets, and wearables has had a positive influence on health, research, and development innovation [17]. More specifically, technology’s impact on clinical research has led to a shift from traditional recruitment strategies to the adoption of new methods [18]. This transition is closely related to the use of advanced technology tools, including social media and web platforms, for patient recruitment [19]. Consequently, a new category of clinical trials, commonly referred to as digital clinical trials, has arisen [20]. These trials use digital recruitment, electronic consent, health data collection formatted in electronic medical records, and advanced analysis methodologies driven by artificial intelligence (AI) [21]. This integration enhances the automation and acceleration of various clinical trial stages [22]. Nowadays, numerous patient-centric digital platforms and applications have been created and introduced with the aim of recruiting and matching participants or patients with the most fitting clinical trials based on their individual profiles, conditions, and needs [23]. Furthermore, certain platforms have a diverse purpose by providing digital recruitment solutions to health care professionals, academic researchers, and clinical trial sponsors involved in clinical research [24]. Several platforms leveraging AI technology are specifically designed to offer an advanced clinical trial matching system [25]. This system enables the seamless integration of patient recruitment into the most suitable trial based on their characteristics and needs [21]. The primary aim of these patient-driven platforms and mobile apps is to facilitate the recruitment process and increase the willingness of patients and healthy volunteers to participate in ongoing clinical studies [26].

One of the latest evolutions in the field of clinical research involves leveraging blockchain technology and AI in health care [27,28]. For instance, blockchain defines and forms a decentralized and distributed digital ledger that records data in the form of transactions across the entire network in a secure and transparent way [29]. Each transaction is recorded in a block, and when the blocks are completed, they are cryptographically linked, creating a chain [30]. Moreover, distributed ledger technology (DLT) is a broader term that includes blockchain technology [31]. However, not all DLTs are blockchain based. Other DLTs include distributed databases maintained by the participants of a distributed network [32]. On the other hand, AI refers to computer systems capable of replicating human behavior and learning to perform tasks through experience [33]. This is accomplished by simulating human cognitive functions, enabling machines to make decisions and perform tasks that typically require human intelligence [34]. Both AI and blockchain technologies share the advantages of immutability, transparency, tamper-resistant records, and no need for a central authority [35]. These technologies offer the capability to improve secure storage and analysis of vast amounts of data, particularly in organizing electronic medical records [36]. As a result, they ensure the highest level of security for handling sensitive datasets [37]. In addition, these technologies ease the process of clinical trial recruitment by offering the prospect to connect, enroll, and allocate appropriate patients into clinical trials matching their requirements on an anonymous basis, securing their personal sensitive information [27]. Therefore, clinical research teams are increasingly focused on choosing the appropriate clinical trial participants according to the settled inclusion and exclusion criteria, leading to the successful conduct of a study as well as protecting participants’ personal sensitive information [38]. In this direction, pharmaceutical companies have turned their investment interest on AI technologies and big data analytics or blockchain as they provide novel solutions by reducing costs and leading the research and development process at the same time toward innovative new paths [39]. Moreover, the COVID-19 outbreak seemed to play a significant role in this transition from manual to virtual clinical trial functions as the remote conduct of clinical trial stages was essential and increased during this period [40,41]. In fact, the COVID-19 pandemic acted as a driving force to simplify the processes of clinical trials through the introduction and development of many important features in electronic format that play a crucial role in clinical trial implementation, such as web-based recruitment, e-consent, and electronic patient matching. These features play a crucial role in clinical trial implementation by improving operational efficiency ensuring benefits for participants [42].

One of the most important principles in medical ethics and the conduct of research following good clinical practice guidelines is the concept of informed consent [43]. Informed consent is required to clearly provide all the details concerning a clinical study to be read carefully and be well understood by all the participants willing to enroll in a clinical trial [44,45]. It serves 2 crucial purposes in clinical research: it promotes and respects participants’ willingness and decision-making regarding enrollment in a clinical trial and also protects individuals from exposure to potential harm [46]. To proceed with the enrollment of participants or patients in clinical trials, written informed consent is an internationally acknowledged requirement [47]. The key elements of informed consent include voluntariness, capacity, disclosure, understanding, and decision [48]. Informed consent in clinical research requires that participants make voluntary decisions by having the capacity to understand well the information provided and be fully informed of all the stages and the aim of the research [49]. Recent studies have further investigated a novel format of this feature called electronic consenting (e-consent). However, it is important to note that electronic consenting is not a new phenomenon. e-Consent has been used in clinical trials for approximately 15 years. Initially adopted slowly, its use has grown significantly due to its benefits in improving patient understanding, enrollment speed, and data accuracy [50]. The term e-consent includes the legally guaranteed participation of patients and volunteers in clinical trials by following accelerated signature processes and avoiding time-consuming submission of files and documents as it is currently used in traditional in-clinic recruitment strategies, known as offline recruitment [51]. The future of clinical research is strongly associated with the use of innovative technologies and novel features that can play an important role in leading to the digitalization of research and development [52].

Another innovative characteristic in clinical trials is the continuous monitoring of patients [3]. It is a critical process that refers to the actual monitoring of patients’ health state and providing health care professionals and researchers with access to important insights and information about patients, such as vital signs, therapeutic response to treatments or medical interventions, and compliance with medical therapeutic schemes, aiming at advanced therapeutic approaches and overall improved patient outcomes [4]. Recent studies have shown that there is a notable dropout rate in clinical trials (30%), raising concerns [22]. Thus, patient monitoring and health care professional counseling can reduce this burden by improving early detection of potential problems and, consequently, minimizing patient dropout rates in ongoing clinical studies [53]. The use of advanced information and communications technologies in combination with novel medical device technologies offers new approaches leading to the development of power-efficient, real-time, and personalized patient-monitoring systems in mobile devices [54]. Technological innovations for advanced measurements like electrocardiography, pulse oximetry, and noninvasive hemoglobin monitoring, as well as the use of smartphones, provide the ability to remotely monitor patients effectively and with an approximate accuracy [55,56]. In summary, the integration of patient health–monitoring features into clinical studies has the potential to significantly elevate the quality and efficiency of the digital tools and innovative approaches designed to conduct clinical trials in a novel digital health care era [17].

### Objectives

The objectives of this study were to (1) provide a comprehensive overview of existing innovative digital tools such as web platforms and applications that focus on patient recruitment; (2) list the tools that also incorporate the continuous monitoring of patients’ health status; (3) illustrate how technology-based interventions can ensure and facilitate the matching of patients to the appropriate trial based on individual characteristics and specific inclusion criteria; (4) present the advantages, challenges, and limitations of these novel technology tools and features in clinical trial recruitment strategies; and (5) highlight the future directions regarding the use of e-technologies and novel features in clinical research.

## Methods

### Study Design

A review with a systematic research approach was conducted. The research team searched the MEDLINE, ScienceDirect, Scopus, and Google Scholar databases to identify relevant studies from January 2000 to October 2024. The search strategy devised for MEDLINE was refined to encompass various combinations of terms related to clinical trials, clinical research, and digital methodologies. Specifically, the search string used was as follows: *{Clinical trials OR Clinical research OR clinical studies} AND e-consent, {Clinical trials OR clinical research OR clinical studies} AND e-matching, {Clinical trials OR clinical research OR clinical studies} AND e-recruitment, Clinical trials AND web platforms, Clinical research AND web platforms, Clinical Research AND mobile applications, Clinical Trials AND artificial intelligence, Clinical Trials AND machine learning*. The aforementioned string was adjusted accordingly for other databases using a combination of Medical Subject Heading algorithms and keywords such as *clinical trials*, *clinical research*, *online recruitment*, *patient matching*, *electronic consent*, and *digital platforms*. Relevant studies were identified and selected through citation searches, as well as by reviewing abstracts, full texts, and peer-reviewed articles.

### Inclusion and Exclusion Criteria

The inclusion of manuscripts in this study was based on the criteria listed in Textbox 1.

**Inclusion and exclusion criteria.**

**Inclusion Criteria**

- Timeline of companies’ operation concerning the digital platforms and tools of interest from 2000 to 2022
- Digital platforms and tools operating internationally without geographical limitations
- Digital tools and platforms that have been scientifically validated through relevant research study publications or peer-reviewed articles in scientific journals
- Digital platforms and tools that retain approval to function, demonstrated through testing and use in clinical trial processes
- Digital platforms and tools with approval from relevant regulatory authorities concerning data safety and transparent function
- Digital tools and platforms in English or with translated websites (and with manuscripts also available in English)
- Digital platforms and tools created by entities from the pharmaceutical industry or academia
- Digital platforms and tools focused on the clinical trial recruitment phase, including features related to patient matching or electronic consent

**Exclusion Criteria**

- Literature reviews
- Digital platforms or tools designed and launched for future testing and operation but not yet validated through scientific publication in peer-reviewed journal

### Study Selection and Data Extraction

In total, 2 independent investigators (AGB and FD-G) initially screened the articles, with any discrepancies resolved through consensus at each step with a third independent investigator (ED). The search results and outcomes were imported into the reference management software Mendeley (Elsevier) for recording, managing, and generating the reference list, including the removal of duplicates. Finally, the findings of the studies were evaluated, summarized, and recorded. All references from the selected studies were retrieved and manually reviewed using the snowball effect [57].

## Results

### Comparison of Platforms for Recruitment

**Figure 1:**
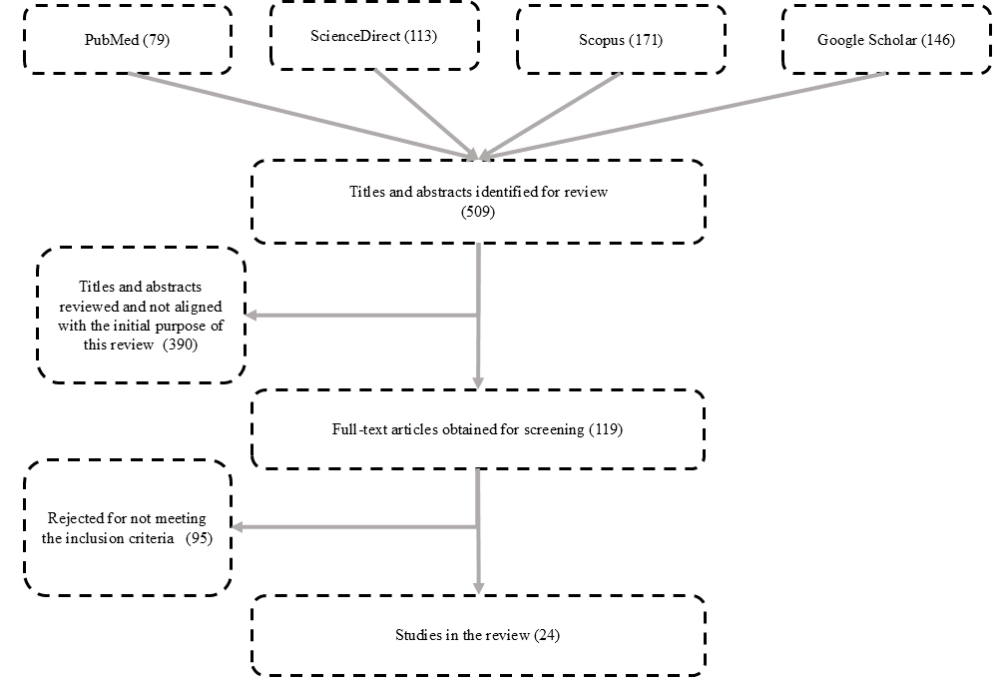
Flow chart showing the study selection procedure.

A flowchart detailing the study selection procedure is presented in Figure 1. Table 1 shows the innovative digital platforms used for clinical trials focusing on the recruitment stage, with the features that they provide, their main characteristics, whom the digital tool is addressed to, and the source from the literature review. These digital tools according to their characteristics were classified into 2 main categories considering the way in which they were launched as solely digital web platforms or mobile apps or web applications. They were also categorized according to whom they were addressed to for use, such as patients or sponsors (eg, health care practitioners, industry professionals, or researchers involved in clinical trial conduct). The key attributes outlined here play a pivotal role in the participant recruitment phase for clinical trials, specifically focusing on the features of electronic consent, web-based recruitment, and patient-matching technologies. In addition, these digital platforms and tools are characterized by several core aspects: the start year of the companies behind these innovations, the total count of patients or users engaged via these technologies, and the geographic presence of each digital tool.

After the classification of the platforms, further investigation and review of the literature was carried out to determine the capabilities of each platform, organize their characteristics into groups, and indicate their pros and cons. The focus was on specific criteria, such as some potential limitations in geographic operation areas, therapeutic fields, approach toward patients or sponsors, educational material availability, patient engagement, scientific evidence, the tools’ ease of use, and financial-related barriers.

Table 2 shows the respective competencies and constraints of each platform identified in Table 1.

**Table 1:** Digital tools supporting the clinical trial recruitment stage and their features and main characteristics.

Name	Type of digital tool	Addressed to patients or sponsors	Main features (e-consent, matching, and e-recruitment)	Platform characteristics (company start year, number of users or patients enrolled, and geographic location)
Accelerated Enrollment Solutions [58]	Digital platform	Sponsors	Matching and e-recruitment	2018; 100-million-patient database (20 million prescreened); United States
Antidote [59]	Digital platform	Patients and sponsors	e-Consent, matching, and e-recruitment	2016; approximately 1 million users; United States
BreastCancerTrials [60]	Digital platform	Patients and sponsors	Matching and e-recruitment	2008; number of users not available; United States
Clara Health [61]	Digital platform	Patients and sponsors	Matching and e-recruitment	2016; 10,000 patients in matching and 50,000 in clinical trials; United States
Clinical Connection [62]	Digital platform	Patients	e-Consent, matching, and e-recruitment	2000; approximately 850,000 patients; United States
Elligo [63]	Digital platform	Sponsors	e-Consent, matching, and e-recruitment	2016; approximately 150 million patients and approximately 1000 studies; United States
Inspire [64]	Application and digital platform	Patients and sponsors	e-Recruitment	2005; approximately 2 million members; United States and India
KU^a^ Cancer Center Clinical Trial Finder [65]	App	Patients	Matching and e-recruitment	2020; approximately 300 app downloads in 2022; United States
Labcorp—Xcellerate Trial Management [66]	Digital platform	Sponsors	e-Recruitment	2011; ≥65,000 patients internationally; United States
MatchMiner [67]	Digital platform	Sponsors	e-Consent, matching, and e-recruitment	2016; approximately 200 patients enrolled; United States
Mendel Health [68]	Digital platform	Patients and sponsors	Matching	2016; approximately 1 million patient health records; United States
ResearchMatch [69]	Digital platform	Patients and sponsors	Matching and e-recruitment	2009; 144,340 volunteers; United States
Trialbee (Hive and Honey) [70]	Digital platform	Patients and sponsors	e-Consent, matching, and e-recruitment	2010; approximately 500,000 patients; Sweden
Trialfacts [71]	Digital platform	Patients and sponsors	Matching and e-recruitment	2006; approximately 20,000 patients and 600 studies; United States

^a^KU: University of Kansas.

**Table 2:** Platforms’ strengths and limitations.

Name	Pros	Cons
Accelerated Enrollment Solutions [58]	Patient-centric approach; strong demographic, psychographic, and behavioral information available Various clinical research therapeutic areas Patient engagement via regular prescreening processes and site follow-ups Telehealth options Education and information available about clinical trials and patients’ treatment options Advanced data modeling system predicting patients most likely to enroll in each clinical trial	A strong presence mainly in the United States but also operating worldwide, with sites in many countries Primarily targeted at sponsors to enhance patient recruitment
Antidote [59]	Patient-centric approach and user-friendly platform design Provision of educational podcasts and webinars Community engagement through encouraging patients to share their health journeys International reach, with operating offices in the United Kingdom and the United States	Despite the educational character on clinical trial awareness, no focus on sharing publicly detailed information on clinical trial outcomes for a more holistic perspective There are clinical trials that are not listed on the platform and may need to use additional resources
BreastCancerTrials [60]	Patient-friendly platform with plain language Clinical trials related to all stages of cancer Emphasis on the rights of patients regarding clinical trial participation; strong medical ethics Provision of educational resources for patients (scientific articles and educational videos)	Geographic limitations as it is a platform primarily focusing on trials available in the United States Focusing mainly on clinical trials of a specific cancer type (breast cancer) A search engine greatly supporting recruitment but not guaranteeing clinical study enrollment, which is particularly crucial for cancer patients, as eligibility often depends on their cancer stage.
Clara Health [61]	Easy to use Patient engagement Focus on public health challenges; supported clinical trials for diagnostics, treatment, and vaccine discovery during the COVID-19 pandemic Personalized support Podcast series about clinical trial experiences	Limited to certain types of medical treatments depending on available partnerships and network Limited integration with other clinical tools or software, such as electronic medical records or laboratory systems May pose some cost barriers according to insurance types
Clinical Connection [62]	Patient-centric approach Telemedicine options Wide range of clinical trials covering a variety of therapeutic areas User-friendly digital environment Options for remote clinical trial participation	Potential geographic limitations; primarily operating in the United States, but there may also be trials extended internationally Limitations in matching of patients with clinical trials based on their personalized characteristics
Elligo [63]	Strong connections with clinical sites and physicians Highly technologically oriented, keeping electronic health records and simplifying clinical trial processes Enhanced data management tools and high-quality services for sponsors	Potential compatibility issues due to advanced technology features in case of concurrent use of other tools by sponsors or researchers Potential financial barriers for some advanced, high-technology services Electronic health record system may pose some privacy concerns for patients or users Limited direct engagement with patients
Inspire [64]	Acceleration and enhancement of enrollment stage Support for the recruitment process by focusing on location, condition, and demographics of participants Educational character, providing information on clinical trial awareness Wide range of trials covering different therapeutic areas Support for patients at decision-making points Community engagement; sharing patients’ and caregivers’ thoughts and experiences Offer of services to sponsors, such as real-world evidence data, patient insights, and digital advertising options Available in the United States and India, offering potential for collaboration with underrepresented and vulnerable populations	Limited description of clinical trial recruitment features; mainly functioning as a patient and caregiver community platform
KU^a^ Cancer Center Clinical Trial Finder [65]	Academic research oriented Prompt allocation of patients to clinical trials “Make a trial referral” feature for HCPs’b and physicians’ ease in their practice A cancer-focused institution Patient support features	Potential geographic limitations due to the operation of the center within the University of Kansas area in the United States Primarily targeted only to patients with cancer, although it is a cancer research institution Mainly available via app format; some restrictions in use
Labcorp—Xcellerate Trial Management [66]	Strong global perspective (≥30 countries involved) Diversity in therapeutic areas Targeted patient recruitment and engagement with the clinical trial process Personalized and user-friendly experience Predictability; identifying potential issues before they occur in a study Medical review evaluation	Potential challenges in integration compatibility with some other electronic systems and software
MatchMiner [67]	Provision of educational material to patients about clinical trials Option for specific genomic profiles related to cancer precision medicine trials Great focus on scientific publications Easy access to the platform without requiring registration or log-in High rates of successful matching Use of open-source AIc technologies	No direct engagement with patients but still facilitates interaction between clinical trial sites and patients Might pose some technology barriers with software complexity for some users with less technical experience
Mendel Health [68]	Outcome validation through AI “intelligent trials” Use of deep learning technology Continuous update of results Focus on real-world evidence analysis and data	Currently focused solely on oncology and populations of patients with cancer Focus primarily on automated eligibility and screening rather than patient recruitment strategies
ResearchMatch [69]	High quality of demographic data and support for diversity of participants Strong scientific impact Option for children registration Flexibility in patient enrollment; no health insurance required	Geographic limitations; only in the United States Mainly focusing on processing the matching stage of a clinical trial; further effort required for the filtering process regarding eligibility criteria for clinical trial participation
Trialbee (Hive and Honey) [70]	Global-oriented platform Focus on patient engagement Patient oriented; enhancing the success of the matching process	Mainly focused on sponsors Some potential limitations in clinical trial options; mainly listing sites or trials that have established partnerships with the platform
Trialfacts [71]	Offers educational resources Promotion of communication or collaboration among researchers Extensive experience in clinical trial management Combination of on-site clinical trials and virtual-based studies Access to a global network of sites and patients Consulting services to researchers on how to avoid recruitment errors	Potential barriers with advanced high-cost services Focusing mainly on sponsors’ needs; not a patient-centric platform but making efforts for more accessible trials for patients and engagement of stakeholders

^a^KU: University of Kansas.

^b^HCP: health care professional.

^c^AI: artificial intelligence.

### Description of the Digital Tools’ Characteristics

Accelerated Enrollment Solutions [58] is a cutting-edge digital platform designed to enhance patient recruitment and increase the successful rate of patient matching in clinical trials. Accelerated Enrollment Solutions offers a diverse range of therapeutic areas and features, including patient engagement, screening processes, location tracking, and telehealth options. The platform also provides advanced data modeling that predicts the likelihood of patient enrollment. However, it should be noted that this digital tool mainly operates in the United States, posing some geographical limitations, and focuses on sponsors (ie, health care providers and researchers) rather than patients.

Antidote [59] is a patient-centered platform that offers e-recruitment, matching, and e-consent features. The platform is easily accessed through web-based searches. One of its characteristics is the emphasis on patient education and information, which is achieved through informative interactive resources such as podcasts, webinars, and videos featuring patient stories. Patients widely accept Antidote for its convenience and user-friendly interface. However, despite the tool’s educational character, there is limited information available regarding clinical trial outcomes for a more holistic comprehension of the clinical study process.

BreastCancerTrials [60] is a user-friendly platform providing features such as e-recruitment and matching in clinical trials focusing on all the stages of breast cancer, prioritizing patient rights, and providing educational content that contributes to patients’ informed decisions regarding their participation in clinical trials. There was no clear evidence of the existence of an electronic consent feature on the interface of BreastCancerTrials. Its main limitations lie in both geographic location (clinical trials conducted mainly in the United States) and type of disease (only breast cancer). In addition, the digital platform system is primarily a search engine and may pose some limitations on up-to-date information, especially for eligibility criteria for clinical trials. Hence, patient enrollment is not guaranteed, which poses a significant barrier for patients with cancer due to challenges concerning their cancer stage and time.

Clara Health [61] is a user-friendly digital tool that has a patient-centric approach providing personalized support. It has released a limited podcast series delving into the clinical trial experience. It is an innovative platform that aims to recruit patients using advanced technology tools and match them to the appropriate clinical trial, although there is no clear information available on whether the e-consent feature is provided by Clara Health. One of its unique features is its focus on public health issues. In the case of the COVID-19 pandemic, Clara Health was very active regarding clinical trials for diagnostics, treatment, and vaccine discovery. Nevertheless, it is worth noting that this platform covers certain types of diseases based on available network partnerships and has limitations in integration with other advanced clinical software, such as electronic medical records or laboratory systems.

Clinical Connection [62] is a digital patient-addressed tool that offers electronic patient recruitment, matching, and electronic informed consent features. It promotes patient engagement and health care provider interaction and enables remote participation in a wide range of clinical trials across various therapeutic areas. Despite its vast potential, Clinical Connection has limited use as it operates primarily in the United States, posing some geographical limitations for patients, but may also offer extended trials internationally. Unlike some other platforms, it lacks a personalized matching function for participants to be promptly allocated to trials based on their personal medical history and preferences.

Elligo [63] as a cutting-edge platform addressed to sponsors for clinical trial processes leverages electronic health records and offers seamless connectivity with clinical sites and physicians, advanced data management capabilities, and high-quality services to sponsors. The platform’s e-recruitment and matching features, as well as its e-consent functions, demonstrate its commitment to streamlining the clinical trial process and enhancing patient engagement. The platform’s vast reach of approximately 150 million patients demonstrates its potential impact on clinical research. However, there are also potential limitations and challenges to this platform, such as compatibility issues with other advanced software and cost barriers due to its high technological orientation. Some patients may also have privacy and security concerns due to the use of electronic health records.

Inspire [64] is a user-friendly digital tool that enrolls patients for clinical trials based on their location, condition, and demographics, covering various therapeutic areas. It offers educational resources and supports patients at crucial decision-making points from diagnosis through treatment. The operations of Inspire in India and the United States enhance collaboration and research with underrepresented and vulnerable populations. Furthermore, it may also present some limitations due to the direct marketing involvement of brand partners on pharmaceutical options and products. Moreover, while it can be used for participant recruitment in clinical trials, it is primarily designed as a patient community platform, which may have some drawbacks compared to other digital tools in the recruitment stage of clinical studies.

University of Kansas Cancer Center Clinical Trial Finder [65] is one of the first observed research-oriented applications that successfully aims to recruit, guide, and match patients with cancer with the most suitable clinical trial for them according to their needs. This platform explicitly states that its target user group includes only patients. The application includes features such as “Make a trial referral” to support health care professionals and physicians in their practice. However, it has certain use limitations as it can be used only as an application and it is addressed also to a specific patient group. Moreover, it may present geographical limitations as it only addresses cancer patients based in the USA. This may also contribute to the limited total number of enrolled patients in the app (300 downloads).

Labcorp’s Xcellerate Trial Management [66] tool is a widely used digital platform that includes >30 countries and targets >50 therapeutic areas. It offers personalized and user-friendly patient recruitment features along with an interactive patient community to direct sponsors (health care professionals and researchers) toward clinical trial success. The platform provides medical review evaluation to ensure patient safety monitoring and identify potential issues before they occur. However, compatibility issues with other advanced technological software may arise. It is important to note that Xcellerate Trial Management primarily focuses on clinical trial management and may not be as effective in other stages of clinical trials, such as patient recruitment or data analysis.

MatchMiner [67] use open AI sources to facilitate patient recruitment for clinical trials, with a special focus on cancer precision trials, including the option to investigate specific genomic profiles, advancing personalized medicine approaches. It also serves as an educational resource for patients, emphasizing as well the publication of scientific articles. The platform is easily accessible, with no registration or log-in requirements, but it does not involve direct patient engagement. MatchMiner offers all the features concerning the recruitment process, such as e-recruitment, matching, and e-consent. There may be platform use barriers and software implementation issues for some users with less technological experience.

Mendel Health [68] uses AI and natural language processing technology to validate its recruitment outcomes through the “intelligent trials” feature, with a focus on analyzing real-world evidence and data. It is the only included platform that offers the matching feature as its main service. Using deep learning technology, the web-based platform offers features that include searching medical literature and patient health records and suggesting evidence-based treatments. Despite its reliance on advanced technology, Mendel Health has a focus solely on oncology and populations of patients with cancer. Its approach is centered on automated eligibility criteria and patient screening strategies rather than on traditional patient recruitment methods.

ResearchMatch [69] is a digital tool that mainly focuses on the recruitment and matching stages of clinical studies. It integrates high-quality demographic data into the recruitment process, ensuring participant and patient diversity, including the incorporation of children as well. Moreover, the platform is financially affordable for researchers and does not require health insurance documentation during patient enrollment. However, it is important to note that, currently, only individuals living in the United States and Puerto Rico have access to this platform, posing geographical limitations. In addition, ResearchMatch excels mainly at the matching stage of clinical trials; hence, researchers need to make organized efforts to filter eligible patients based on study-specific inclusion criteria. It is not clearly indicated whether any type of electronic consent is provided through this tool.

Trialbee (Hive and Honey) [70] is a patient-centric platform that possesses all 3 features concerning the recruitment process (e-recruitment, matching, and e-consent). With a global reach that leverages patient information, Trialbee endeavors to improve the matching process. While it targets patient needs, Trialbee primarily serves health care providers and researchers, mainly referring to sites or trials with whom it has established partnerships, potentially excluding other relevant clinical trials available.

Trialfacts [71] is a comprehensive platform that focuses on the preparatory stages of conducting a clinical study, emphasizing patient recruitment and matching processes. One of its primary objectives is to combine on-site clinical trials with virtual-based studies. The platform provides valuable educational resources and promotes strong interaction among researchers. With extensive experience in clinical trial management, Trialfacts offers access to a global network of sites and patients. It provides consulting services to researchers to avoid recruitment errors. However, it does not guarantee clinical trial enrollment, and the platform lacks direct patient outreach as a function.

### Comparison of Platforms for Recruitment That Emphasize Monitoring Features

Table 3 shows digital tools that additionally provide the function of participant or patient monitoring. Their main features, characteristics, and the enrolled patients and users are shown. Monitoring entails tracking the patients’ health status (compliance with treatments or interventions and vital sign measurements) during the trial and following its completion. The vital signs of patients are recorded through wearable sensors, also linked with other fitness- or health-tracking apps, and online diaries that focus on medication adherence. These tools enable health care professionals and researchers to monitor patients’ progress accurately, identify potential adverse effects, and assess the effectiveness of the intervention in a clinical study. The monitoring features of these digital platforms are crucial in ensuring the safety and well-being of patients during and following a clinical study, contributing to the overall success of a clinical trial.

**Table 3:** Digital tools facilitating the recruitment stage of clinical trials that incorporate the feature of monitoring patients’ health status.

Name	Type of digital tool	Addressed to patients or sponsors	Main features (e-consent, matching, and e-recruitment)	Platform characteristics (company start date, number of users and patients enrolled, and geographic location)
Advarra [72]	Digital platform	Sponsors	e-Consent and e-recruitment	2020; approximately 56,000 participants; United States
Castor [73]	Application and digital platform	Patients and sponsors	e-Consent, matching, and e-recruitment	2012; approximately 7 million patients and 147,000 users; United States and EU^a^ (Amsterdam)
Clinical Ink [74]	Application and digital platform	Sponsors	e-Consent and e-recruitment	2020; approximately 2 million patients; United States
Clinical Research IO [75]	Digital platform	Sponsors	e-Consent and e-recruitment	2015; approximately 7 million patients; United States
Clinpal [76]	Application and digital platform	Sponsors	e-Consent, matching, and e-recruitment	2012; approximately 10,000 patients; United Kingdom
Spark [77]	Digital platform	Sponsors	e-Consent and e-recruitment	2009; number of users and patients not available; United States
Eureka [78]	Application and digital platform	Patients and sponsors	e-Consent and e-recruitment	2018; 83,000 participants (during the COVID-19 pandemic); United States
Massive Bio [79]	Application and digital platform	Patients and sponsors	Matching and e-recruitment	2015; ≥100,000 patients; United States
Medable [80]	Digital platform	Sponsors	e-Consent, matching, and e-recruitment	2012; approximately 1 million patients; United States
Veeva Systems [81]	Application and digital platform	Patients and sponsors	e-Consent, matching, and e-recruitment	2007; number of users and patients not available; United States

^a^EU: European Union.

Table 4 shows the respective competencies and constraints of each platform identified in Table 3.

**Table 4:** Platforms’ capabilities and constraints.

Name	Pros	Cons
Advarra [72]	Telehealth, remote data collection, and virtual patient visits Enhanced engagement through mobile-compatible tools for remote participation	No information about patient-matching feature, potentially limiting recruitment capabilities
Castor [73]	A robust e-consent system and participant recruitment process User-friendly electronic data capture Support for decentralized clinical trials with tools for remote patient monitoring and ePROsa Strong patient engagement (>7 million patients and 147,000 users) Global presence	May require additional integration of tools for trial management New users may face barriers with more advanced features
Clinical Ink [74]	Advanced e-consent and e-recruitment Support for decentralized and hybrid clinical trial real-time data capture; advanced monitoring via sensors, wearables, and televisits Strong focus on patient engagement	Limited information on matching features Technology complexity Potential connectivity issues due to cloud-based functionalities Primarily patient focused; further tools may be needed for sponsors Limited global reach; mainly in the United States
Clinical Research IO [75]	A robust participant recruitment process, simplifying enrollment Support for decentralized clinical trials with tools for remote patient monitoring and ePROs Advanced patient-matching tools and comprehensive monitoring Strong patient engagement	More focused on sponsors Limitations in financial tracking–related issues for clinical trial conduct
Clinpal [76]	Provision of learning and advanced training mechanisms for researchers Suitable for remote (virtual), hybrid, and direct-to-patient clinical studies Cloud-based software as a service platform Easy-to-use platform Real-time compliance or performance metrics through the integration of external technology devices Support for patients in the overall clinical trial management process with engagement and reminder mechanisms	More focused on sponsors The app is not widely used Potential technical limitations
Spark [77]	Limitations in financial tracking–related issues for clinical trial conduct	More focused on sponsors Limited detailed information on specific patient-matching features Limited updated information on clinical trial outcomes and number of users enrolled
Eureka [78]	Academic research oriented Remote monitoring Creation of electronic medical records Focus on rare diseases and minority populations Active research during public health crises such as the COVID-19 pandemic Global perspective; involving international patients Provision of personalized health services Strong publication record on high-impact scientific journals Educational material and podcasts available	Matching feature not clearly described Potential technical limitations
Massive Bio [79]	Strong engaging community between patients and health care professionals A digital environment for personalized and precision medicine solutions Compliance with and security certifications from regulatory authorities (eg, EMAb) AIc advanced technology for the matching stage (patient prescreening) and data analysis	Geographic limitations; mainly in the United States Offer of clinical trials for patients with cancer; no variety regarding other diseases Potential barriers due to costs
Medable [80]	Comprehensive e-consent solution Efficient recruitment and patient matching Integration with wearables and sensors Patient engagement features	More focused on oncology and vaccines
Veeva Systems [81]	Strong global reach; operates in key regions worldwide Robust patient engagement tools Integration flexibility with other clinical trial management systems Decentralized clinical trial support via advanced e-recruitment	Services and advanced tools that may be challenging; potential complexity in navigation for new users Potential financial barriers

^a^ePRO: electronic patient-reported outcome.

^b^EMA: European Medicines Agency.

^c^AI: artificial intelligence.

### Description of the Characteristics of the Digital Tools That Emphasize Monitoring Features

The Advarra [72] platform is a comprehensive digital tool designed to support decentralized clinical trials. It provides a wide range of features, including e-consent, telehealth, remote data collection, and virtual patient visits, enabling clinical trials to be conducted seamlessly across multiple locations. These capabilities help improve engagement with participants and streamline trial management for stakeholders. However, a limitation of the platform is that it does not include a dedicated patient-matching feature, which could hinder recruitment efficiency in certain studies.

Castor [73] is a comprehensive platform dedicated to clinical trial management, offering advanced features for e-consent and patient recruitment and monitoring. It is a useful tool for sponsors and patients. According to its records, it may support >7 million patients and 147,000 users. Its tools offer advanced support for decentralized trials, patient matching, electronic patient-reported outcomes, and remote monitoring. However, while Castor excels in patient engagement and data capture, more advanced tracking activities may require integration with other tools. It has a global presence, with offices in New York and the European Union (Amsterdam), providing reliable support for trials across multiple regions.

Clinical Ink [74] is a robust clinical trial platform with advanced features in patient recruitment, engagement, and monitoring with cutting-edge technology such as e-consent, sensors, wearables, and televisits. Despite being a relatively new platform, it serves approximately 2 million patients. The platform’s strengths are optimizing patient experience, supporting decentralized clinical trials, and enabling real-time data collection through wearables and sensors. It has a patient-centered approach, but it may require some additional tools for overall clinical trial management and especially for sponsors. This platform shows a great potential for supporting clinical studies, although it is primarily available in the United States, posing some geographic limitations.

Clinical Research IO (CRIO) [75] is a comprehensive digital platform designed to enhance the management and execution of clinical trials. The platform offers a wide array of features, including centralized patient recruitment, advanced e-consent forms, and tools that foster strong patient monitoring. These capabilities help streamline operations and improve data quality for single sites, sponsors, and academic partners. While CRIO excels at simplifying workflows for these stakeholders, there are limited references to patient-matching features. Despite this, CRIO remains a valuable solution for clinical trial management.

Clinpal [76] is a digital tool that is accessible on web and mobile platforms, providing e-consent and health-monitoring features in addition to its other necessary existing functions for clinical studies. It tracks real-time compliance with treatments or interventions but also performance metrics and integrates with external devices for clinical trials. It also offers remote, hybrid, and direct-to-patient clinical study participation through a cloud-based software platform and assists patients with engagement and reminders for the overall clinical trial process. Clinpal focuses mainly on researchers and health care providers. Relative bias may occur due to the nature of self-monitoring, the sensitivity of wearable sensor measurements, and the reliance on self-reports from patients.

DrugDev’s Spark [77] platform offers a unified suite of solutions aimed at optimizing clinical trial operations. Its key features include e-consent, site selection tools, and data-tracking tools such as eTrackers that automate patient retention, enrollment diversity monitoring, and trial management processes. There is limited transparency on the exact number of patients enrolled through the platform, and it is unclear whether the platform provides specific patient-matching features.

Eureka [78] is an academic-oriented platform that aims to enhance clinical studies of rare diseases in minority populations. This platform has been actively facing public health challenges such as the case of the COVID-19 pandemic, with their research involving approximately 83,000 participants (enrolled number). It aims to enhance patient recruitment stages and provides e-consent features to accelerate enrollment processes. It also supports the remote monitoring of clinical trial participants or patients, data collection from wearables and apps, and the creation of electronic health records, advancing a personalized medicine approach to clinical trials. Eureka has an impressive record of publications in high-impact scientific journals and available educational material and podcasts concerning clinical trials. However, the recruitment process’s efficacy may have limitations in some aspects due to the unclear statement of the matching feature.

Massive Bio [79] is a commonly used e-recruitment and matching digital tool with a considerably large number of participants. It uses AI technology to assess patient data, identify individuals who meet the eligibility criteria for specific clinical trials, and conduct comprehensive data analysis to streamline the recruitment process. It provides personalized medicine and a precise environment for patients, including the exchange of patient experiences and their journeys through its Patient Ambassador Program. Massive Bio is compliant with and certified by regulatory authorities such as the European Medicines Agency, but it is solely available to the US population and targets patients with cancer.

The Medable [80] platform is a unified, cloud-based solution designed to support decentralized, hybrid, and traditional clinical trials. It offers a comprehensive set of features such as e-consent, electronic clinical outcome assessments, virtual patient visits, and e-recruitment. However, while the platform supports recruitment indirectly, it does not offer a dedicated patient-matching feature, which may limit its recruitment capabilities for some studies.

Veeva Systems [81] is a comprehensive platform for clinical trials with a strong global presence in different continents, such as North and South America, Europe, and Asia, offering a variety of tools for e-recruitment, patient engagement, and decentralized clinical trial support. However, although Veeva Systems is rich in features, it can also be quite complex in navigation, requiring more advanced use, particularly in the case of e-consent. Its integration capabilities and global reach make it ideal for large-scale multicenter trials, though its cost can present a potential challenge

## Discussion

In this section, our study findings, strengths, and limitations are discussed. In addition, concept technological solutions are presented based on blockchain and AI, along with the perspectives that support them.

### Comparison of the Digital Platforms

The platforms presented in this study and their features refer to a specific time frame when the investigation took place. According to our findings, there is an adequate number of digital platforms (N=24) focused on orienting the early stages of clinical trials, specifically focusing on matching, e-consent, and patient recruitment. Most of these digital platforms (n=22, 80%) are headquartered in the United States, but most of them (n=18, 75%) also provide global access to their features, whereas 25% (n=6) primarily provide services within the United States. These platforms attempt to provide a well-informed digital environment for patients and simplify the clinical trial processes. This review revealed that 8% (n=2) of the platforms have enrolled >100 million participants, 29% (n=7) have >1 million participants, 4% (n=1) have approximately 200 participants, and participant numbers were unclear for 17% (n=4) of the platforms. Outcomes indicated that these digital platforms and applications have an adequate number of participants, representing an acceptable sample size, also assisting in the successful conduct of clinical trials in rare diseases. It was also shown that many of the platforms (n=14, 60%) have clearly incorporated an electronic consent form as a feature that promotes autonomous decision-making and simultaneously protects human rights. In addition, most of the digital platforms seem to serve different purposes and scopes for both researchers or sponsors and patients. More specifically, 8% (n=2) of them leverage patient demographics to achieve an accurate match of patients with clinical studies, whereas 38% (n=9) of the platforms offer sponsors or researchers a clinical trial management plan for the conduct of their research. Furthermore, 50% (n=12) of the platforms offer dual functionality, addressing the needs of both patients and sponsors, providing a more integrated approach to managing clinical trials. Most of the platforms (n=10, 42%) incorporate educational features, providing valuable information to patients and explaining the purpose of clinical trials, encouraging the interaction and exchange of ideas in the patient community. The platforms differed in scope, with 21% (n=5) focusing primarily on oncology, whereas others addressed broader populations. The flourishing and increasing use of such digital tools appeared to arise since the COVID-19 outbreak, when the barriers that were created due to the lockdown restrictions posed a great burden to the efficient conduct of clinical studies. In contrast, while these digital tools may offer multiple benefits for a digital new era in clinical trials, such as increased patient participation, faster progression through clinical trial stages, and personalized monitoring features there are also many challenges. Accessibility, compatibility, and accuracy are just a few examples of issues that can arise. Older individuals and those unfamiliar with newer technologies may have limited access to and use of these tools. Furthermore, due to the prevalence of the development, design, and availability of these digital tools in specific geographic regions such as the United States, cultural and language barriers may arise and limit the access and use of these technologies in other regions of the world. Another challenge is the potential for transparency issues if e-consent features are unclear and not accompanied by detailed information on all patient rights. Finally, it is important to note that integrating telehealth options into some of these clinical research tools entails risks, including the potential for invalidity as self-monitoring systems still need improvement. By considering carefully the integration of innovative digital tools into clinical research, researchers can maximize the benefits of these technologies while minimizing the risks and challenges associated with their use.

### Novel Technologies (AI Based) and Software Underlying the Development and Functioning of Innovative Digital Platforms Contributing to Clinical Research

Advancements in digital health tools and emerging technologies play a crucial role in revolutionizing the design, development, and operation of systems supporting the clinical trial recruitment process, thereby significantly enhancing the value of these systems within the clinical research domain. Blockchain or DLT and AI combined can provide the best technical elements of both worlds. Sponsors, clinicians, and researchers can make decisions with greater ease owing to machine learning models, which can analyze massive amounts of data and offer sufficient decision-making tools. For instance, finding the best candidates and recruiting patients are crucial steps in the clinical research process. AI and machine learning reduce the time and cost of identifying these patients by analyzing both structured and unstructured electronic health record data. For example, in a clinical trial, the sponsors can send a matching request to the blockchain system, which, supported by AI, can automatically match the potential patients, saving time for principal investigators, researchers, and patients. The blockchain or DLTs can ensure the validity of a clinical trial and the sponsor’s identity. In this context, patient identification speeds up and leads to improved and more accessible and efficient clinical trials [82]. On the other hand, finding the best researchers and candidates that can lead and perform a clinical trial is also a time-consuming issue that may be accelerated using deep learning techniques [83]. Identifying highly skilled and experienced investigators is crucial for successful drug development, implementing cost-effective strategies, and selecting the most appropriate patient candidates for clinical trials. Combining these values from the AI domain with the security, privacy, and transparency features of blockchain leads to advanced clinical research systems able to produce robust data management in clinical trials. Modern applications using DLTs offer maximum data management and security capabilities. More specifically, clinical trial data that are recorded on the blockchain are protected against unauthorized access and alterations or tampering. In addition, DLT features such as decentralization, immutability, data provenance, and auditability increase trust and awareness. The highest level of trust between software and users (eg, patients, physicians, sponsors, and researchers) can be achieved by relying on the data recorded in blockchain. Data integrity through transparency reinforces the trust among all the stakeholders involved in a clinical trial process [84]. Other values that blockchain contributes to clinical trial tasks are data management, analysis and reporting, patient privacy, patient retention, and regulatory compliance. Solving the problem of trust for the entire clinical trial ecosystem fosters seamless collaboration among stakeholders and people willing to collaborate easily, trusting the system in a decentralized way rather than trusting a centralized system owned by one entity. Most individuals are willing to share their medical data anonymously if privacy and security are guaranteed [85]. Community-driven clinical trials represent a new era, characterized by trusted environments, privacy by design, voluntary participation, user-controlled data sharing, anonymized raw data, secondary studies, and metadata analysis. These pillars are supported by DLT, enabling secure and transparent processes. Nonetheless, ensuring the ethical exploitation of AI and blockchain tools is yet another priority to address when using such technologies. In summary, the collaboration among AI, blockchain, and other digital health platforms able to support clinical research can reduce time-costly procedures and bureaucracy burdens, provide privacy and data control, and empower participants to be more active.

### The PharmaLedger Innovative Web Platform and PharmaLedger Association

PharmaLedger is an example of how blockchain technology can be applied in various use cases of the health care domain. It has revolutionized the way data are handled and stored in ledgers and how privacy and data ownership are controlled in a decentralized way. PharmaLedger [86], as a European Union and Innovative Medicines Initiative project, exploited this blockchain technology to bring together experts in technical, legal, and regulatory fields, as well as pharmaceutical companies and patient organizations to accelerate the innovations in health care and benefit specifically the patients and the entire health care ecosystems. The overall target of the project was to leverage blockchain technology to address critical points of friction, fraud, and waste management in pharmaceutical development and distribution. The project developed several use cases in the pharmaceutical and health domains:

- e-Leaflet or electronic product information. This serves as the digital counterpart to traditional paper leaflets in medicine, offering enhanced traceability of the product, data integrity, interoperability, and patient empowerment.
- Supply chain management. Applying blockchain technology provides many benefits, such as traceability of medical products in a tamper-resistant way, authentication and anticounterfeiting, data integrity, efficient recalls, and smart payments.
- Clinical trial recruitment. This use case addressed many challenges, such as time-costly patient recruitment procedures, enrollment times, financial problems, and accessibility and diversity issues.
- Internet of Things and personalized medicine. This approach collects data from medical devices of patients who agree to participate in a clinical trial in an automated way, stores them in the ledger in encrypted ways, and gives the user control to let their data be used by other researchers. These cases exploit a novel dynamic permission algorithm that provides real-time permission to researchers to perform secondary studies. Patients can see how their data are used, and they can revoke permission at any time and may withdraw their participation completely.
- e-Consent. Electronic consent is for certain applications in clinical research, to create value; work in a digital way; and provide immutability, digital signatures, and all the essential elements required for modern platforms of clinical research.

The core architecture behind the PharmaLedger project is an open-source platform called OpenDSU [87]. The PharmaLedger project is considered a blockchain of blockchains network or a multiblockchain architecture (hierarchical) and is mainly characterized as a blockchain-agnostic network. In other words, using OpenDSU helped create a big ecosystem of trust that can work with any other permission or public blockchain. Most of the use cases required off-chain data management due to the limitations of the transaction size in the ledger, the complexity of the smart contracts, the execution time, the finality time, and the cost of writing data to the ledger. To avoid these problems, OpenDSU provides an extra layer of transactions that separates the off-chain data from the on-chain data and maintains only the essential transactions with their hashes on the ledger. Security, privacy, and confidentiality are embedded into the architecture with user wallets and client-side encryption along the self-sovereign applications. Providing a flexible architecture and keeping it blockchain agnostic creates valuable support for all the different use cases. PharmaLedger uses key design principles, such as (1) hierarchical blockchain structure, (2) blockchain agnosticism, (3) code integrity, (4) use case flexibility, (5) efficient smart contract use, and (6) decentralized identities and flexible deployment procedures, among others. As previously mentioned, data-sharing units (DSUs) can be thought of as standard entities in computer science, but they are encrypted. However, they can converse, and they carry arbitrary code and data as well. Each DSU is then divided into many smaller parts called “bricks,” and they form a brick map that has the links to all bricks. A person reconstructs the DSU object from the brick map when interacting with a DSU. Because no one can control it, anyone can host DSUs anywhere and at any time without worrying about privacy. The code and data are only visible to the owners. Finally, the PharmaLedger Association (PLA) is the continuation of the successful PharmaLedger project [88]. It evolved as a nonprofit association to deliver the Digital Trust Ecosystem in Healthcare. The focus is being shifted to the patients following the principles of PharmaLedger, such as neutrality, inclusivity, simplicity in coding, transparency, and open source. The main lines of development and research are decentralized trials, product trust, and supply chain. Many companies from the public to the private sector have already joined the association and exploited this new ecosystem. Previous use cases from the PharmaLedger project were transferred to the PLA XLAB, which stands for the innovation arm of the PLA and allows for the incubation and development of prototypes and demonstrators.

### The Future of Novel Technology Digital Tools in Clinical Research and What to Expect

The future of emerging technologies in clinical research will witness transformative advancements driven by AI, blockchain, and innovative tools that are able to provide personalized solutions to all the stakeholders involved in the health care domain. The algorithms based on AI are revolutionizing the identification of suitable candidates and reducing the costs and time required for such procedures. The integration of blockchain into these technologies is a good fit for clinical trials and especially for remote clinical trials. These technologies not only maximize security but also enhance the privacy elements that were missing up to this point and ensure the transparency of clinical trials. Data security is ensured through the trial processes and transactions, as well as the accuracy and integrity of data exchanged among stakeholders during the clinical trial. The blockchain can provide improved data management and real-time analysis with the actual consent of patients, enabling patient empowerment and assured decisions. With new user-friendly interfaces, mobile apps, and other novel technology mechanisms involving blockchain, patient engagement will be advanced even further. Transparency, privacy, and ethical considerations will be the primary forces to be considered when creating such solutions. Better clinical research instruments and prompt trial schedules are anticipated in the future, which will promote patients’ comfort and provide them with better clinical trial experiences. The future of clinical research will be shaped by the integration of AI systems, such as chatbots, virtual assistants, predictive analytics, and data-driven decision-making tools, which will redefine the current tools in use. A new era is dawning on the conduct of clinical trials characterized by heightened standards and cutting-edge technologies. This shift contributes to the ongoing expansion, progression, and advancement of this field.

### Strengths and Limitations of This Study

Ours is one of the first studies conducted at a European level reviewing and providing valuable information on innovative digital platforms available internationally focusing on features that enhance and accelerate complex clinical research processes such as the clinical trial recruitment stage. The review and selection of the studies was conducted with a careful systematic approach and validated by a team of researchers. One researcher conducted study collection; the other 2 reviewed the studies; and they all conducted the final screening, selection, and decision-making regarding the platforms included in the final tables. Concerning limitations, there were many digital platforms available on the internet that did not match the inclusion criteria for this review. One of the main issues was that many novel platforms had not been reviewed or tested enough in real time by researchers or had not been validated via scientific reports or published studies.

### Conclusions

This study highlights the pivotal role of the identified innovative digital platforms in clinical trials, contributing significantly to recruitment efficacy, patient engagement enhancement, trial stage acceleration, and personalized patient vital sign monitoring. The positive impact of these platforms underscores their potential to revolutionize the landscape of clinical research. However, many challenges remain, such as issues of accessibility, digital compatibility constraints, and transparency issues that necessitate careful consideration of these tools’ use. Addressing these challenges is not only vital for the seamless integration of digital tools into clinical trials but is also crucial for ensuring the conduct of trials following all the appropriate ethical considerations. Achieving a balance between innovation and ethics is essential to optimize the benefits derived from digital tools while effectively managing the associated risks. Future advancements in digital technology and ongoing efforts to address challenges are poised to further enhance the efficiency and ethical standards of clinical trials, ultimately advancing medical research and patient care.

## Abbreviations

AI: artificial intelligence
CRIO: Clinical Research IO
DLT: distributed ledger technology
DSU: data-sharing unit
PLA: PharmaLedger Association
